# Evaluation of an interactive case simulation system in dermatology and venereology for medical students

**DOI:** 10.1186/1472-6920-6-40

**Published:** 2006-08-14

**Authors:** Carl-Fredrik Wahlgren, Samuel Edelbring, Uno Fors, Hans Hindbeck, Mona Ståhle

**Affiliations:** 1 Dermatology & Venereology Unit, Department of Medicine, Karolinska University Hospital Solna, Karolinska Institutet, Stockholm, Sweden; 2 Department of Learning, Informatics, Management and Ethics (LIME), Karolinska Institutet, Stockholm, Sweden

## Abstract

**Background:**

Most of the many computer resources used in clinical teaching of dermatology and venereology for medical undergraduates are information-oriented and focus mostly on finding a "correct" multiple-choice alternative or free-text answer. We wanted to create an interactive computer program, which facilitates not only factual recall but also clinical reasoning.

**Methods:**

Through continuous interaction with students, a new computerised interactive case simulation system, NUDOV, was developed. It is based on authentic cases and contains images of real patients, actors and healthcare providers. The student selects a patient and proposes questions for medical history, examines the skin, and suggests investigations, diagnosis, differential diagnoses and further management. Feedback is given by comparing the user's own suggestions with those of a specialist. In addition, a log file of the student's actions is recorded. The program includes a large number of images, video clips and Internet links. It was evaluated with a student questionnaire and by randomising medical students to conventional teaching (n = 85) or conventional teaching plus NUDOV (n = 31) and comparing the results of the two groups in a final written examination.

**Results:**

The questionnaire showed that 90% of the NUDOV students stated that the program facilitated their learning to a large/very large extent, and 71% reported that extensive working with authentic computerised cases made it easier to understand and learn about diseases and their management. The layout, user-friendliness and feedback concept were judged as good/very good by 87%, 97%, and 100%, respectively. Log files revealed that the students, in general, worked with each case for 60–90 min. However, the intervention group did not score significantly better than the control group in the written examination.

**Conclusion:**

We created a computerised case simulation program allowing students to manage patients in a non-linear format supporting the clinical reasoning process. The student gets feedback through comparison with a specialist, eliminating the need for external scoring or correction. The model also permits discussion of case processing, since all transactions are stored in a log file. The program was highly appreciated by the students, but did not significantly improve their performance in the written final examination.

## Background

Approximately 5–15% of patients in general practice seek advice because of symptoms and signs from the skin [1-3] or suspicion of sexually-transmitted infections. It is therefore very important for medical students to achieve basic knowledge of and skills in the most common and important disorders in dermatology and venereology. In general, this topic is dealt with in a 3-to-4-week undergraduate course in the Swedish medical programmes. The course usually includes traditional lectures, seminars/workshops, bedside teaching and outpatient clinics. Some universities give priority to problem-based learning, whereas others, including our unit, have a mixed teaching approach using various forms of student-centred educational methods.

Numerous computer-assisted learning (CAL) programs have been introduced in medical education. In fact, reports on such programs began to appear in the late 1960s (e.g. [4]). Over this period, the technology has evolved from mainframe computers to multimedia microcomputers and Internet access. The visual basis of dermatology and venereology makes the application of CAL attractive. Consequently many computerised programs and Internet sites are available in this speciality, offering for example lecture notes, clinical and dermatopathological images (e.g. [5]), quizzes, extensive atlases (e.g. [6,7]) and elaborate interactive case-based study (e.g. [8,9]). However, the educational value of CAL in dermatology and venereology undergraduate teaching has rarely been evaluated in terms of learning outcomes. One exception is the German "Practical Training Programme Dermatology 2000", which improved students' knowledge and skills [8].

The existing dermatology and venereology packages were considered unsuitable for our students, since we wanted them to practise taking patient's histories, writing medical records and carrying out other parts of the clinical process, requiring a simulation program in Swedish. In addition, the pedagogical approach of most available programs was considered less suitable for our purposes.

The present aim was to present the development and to study the effect of a new Swedish interactive computerised teaching program in dermatology and venereology (NUDOV), primarily intended for medical-school undergraduates. NUDOV is an acronym for Nationellt Undervisningsprogram i Dermatologi Och Venereologi; i.e. national educational system in dermatology and venereology.

## Methods

### Phase I: Development of the concept and prototype

The first project year was spent developing a user-friendly and flexible interface framework, based on an authentic pilot case. Through interaction with test students, teachers in dermatology and venereology and other medical-educational expertise, a model gradually emerged.

**Figure 1:**
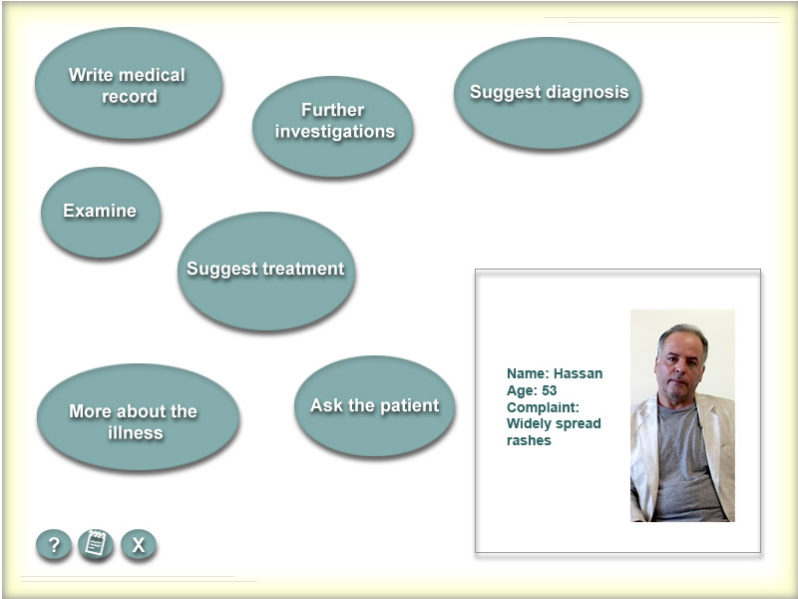
User's options (menu) for the management of a patient. The text is in Swedish in the program.

**Figure 2:**
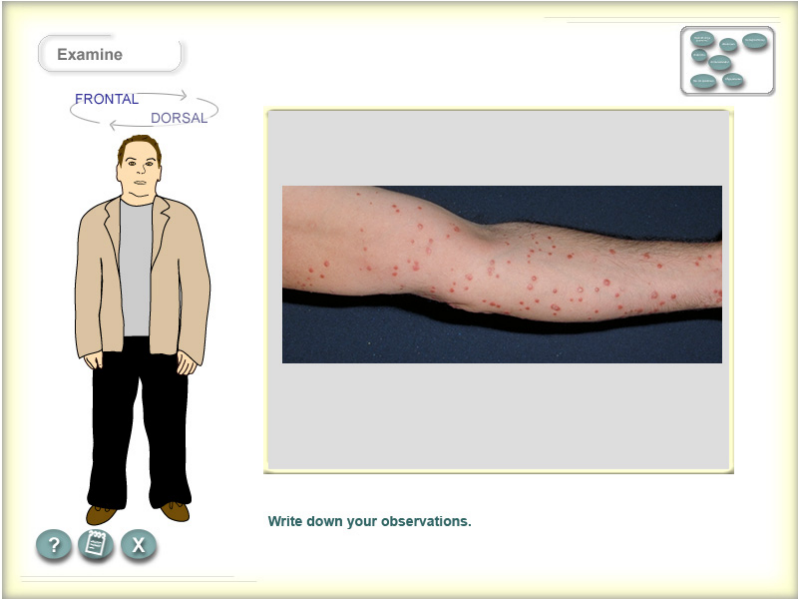
The skin of a patient can be examined by clicking on a cartoon that can be rotated. Images can be magnified. The text is in Swedish in the program.

The user selects a patient in a virtual waiting room and then manages the patient. The following options can be chosen in any order (Fig. 1): suggest questions for medical history, examine the skin (Fig. 2), and suggest further investigations, diagnosis, differential diagnoses and treatment, and writing a medical record. Regardless of which patient is chosen, the same interface meets the user. Patient-specific data is unique for each case and consists of text, images and video clips. When the student has suggested a diagnosis and differential diagnoses, he/she may enter a section called "More about the disease", giving extensive information with a large number of images including differential diagnoses and links to clinical and scientific Internet sites. The concept includes the possibility for the student to: (1) work with authentic patients at his/her own pace, (2) investigate the patients without having to follow a strict point-by-point protocol, (3) write prescriptions and patient records, (4) obtain feedback by comparing his/her own work with that of an experienced colleague (a specialist), and (5) perform a process analysis of the work with each patient, since all transactions within the program are stored in a log file. To be able to retrieve the expert opinion on the various matters, like diagnosis, treatment etc., the student has to enter his/her own suggestions.

### Underlying pedagogical considerations

To stimulate student activation and reflection the interactions in NUDOV are designed in a problem-oriented manner where the student leads the investigation and management of the virtual patient. The open structure allows different lines of reasoning, both to reach a diagnosis and in management of the case. So while students mostly arrive at the same diagnosis and roughly the same treatment, they vary in their process of arriving there. To allow individual variation, there is no "one and only" correct way marked out by the software. Instead the student compares his/her actions with those of an expert. This is done when students actively ask for this feedback and is not steered by the system. The expert's actions are normative and correct, but not "the only truth". Consequently, the student is not corrected or scored by the computer, which is an important difference from verification feedback such as "correct" or "wrong answer, try again".

Modern research on the clinical reasoning process and development of expertise asserts that experts develop cognitive instances of typical cases, "illness scripts", and rely on contextual factors in developing and using these [10]. Taking this into account, the case presentations were developed with rich contextual facts for each patient. Another reason to keep contextual factors in the patient presentation is to minimise the risk of depersonalising the students' conception of the case [11]. Students can now anchor the diagnosis to an authentic person. We expect the self-directed usage, the rich presentations and authenticity to support a meaning-oriented approach to learning, which has proven to correlate well with study success for advanced medical students [12].

### Technical solutions

The authoring tool Macromedia Authorware 5.2 was used for the overall computer programming of the NUDOV framework. To enhance functionality, additional Windows programming was done using complementary tools. A user's notebook was developed. Video clips were edited and compressed with Microsoft mpeg4 codec. Sound was compressed with mp3 codec. Even though the Authorware authoring system allows the content to be run on the Internet, this was not used in the current version of NUDOV, mostly because of the frequent use of video clips and the download time for these. Although informed consent was obtained from all patients, there was still some hesitation concerning web delivery due to ethical reasons. Other reasons against web delivery included the bandwidth problem with the amount of data involved. Therefore, CD-ROM packaging was decided upon. The CD contains an installation file, which installs the application on an ordinary pc equipped with a sound card.

### Phase II: Collection of cases and further feedback from students

During the following two years, typical and authentic cases were recruited. In a venereology case, however, an actor was used. The cases included detailed photographing and video tape-recording of histories and certain practical diagnostic and therapeutic procedures (e.g. "direct microscopy", Doppler sound investigation, taking of skin biopsy specimens, minor skin surgery). In addition, a new extensive digital image archive with differential diagnoses from several hundred patients was collected and organised.

Small groups of students participating in the dermatology and venereology course were recruited to test the new program as it emerged. The students' previous computer experience and "addiction" ranged between diminutive and extensive. Through regular discussions and training sessions, extensive feedback was obtained from the students, enabling us to address didactic problems and to adjust inconsistencies and technical problems.

### Phase III: Small-scale implementation and evaluation

The program was evaluated in a prospective study of undergraduates from three consecutive 17-day-courses in dermatology & venereology in the seventh term of the medical programme at Karolinska Institutet. A course administrator randomly assigned the students to one of two study groups: one with conventional teaching (i.e. lectures, seminars/workshops, bed-side teaching, out-patient clinics) without access to NUDOV (non-NUDOV group; n = 85), and another with conventional teaching plus NUDOV (NUDOV-group; n = 31; two 4 h-sessions). This study design was chosen because NUDOV is intended to be a complement to the existing course structure. The NUDOV group had to be smaller for logistical reasons, because a learning laboratory outside the hospital containing only 12 computers had to be used in order to guarantee that no mixing occurred between the two groups. Consequently, no CDs were distributed to any student. There were no significant differences in age or gender between the two study groups. No student complained about the randomisation or objected to participating.

NUDOV was evaluated subjectively with a student questionnaire, completed prior to the course examination. For rating of the program, the students used a semiquantitative 4-stepped scale (e.g. very bad, bad, good, very good). In addition, comparing the scores of the NUDOV and non-NUDOV groups on the final written dermatology and venereology examination made an objective evaluation of the putative impact of NUDOV. A teacher working in a different hospital without any information about NUDOV always constructed the examination, which was assessed under coded conditions.

The examination comprised two sections with 20 questions each, resulting in a possible maximum of 80 points (= 100%). In the first section, the student should give the most likely diagnosis for 20 photographed clinical cases. The second section consisted of short answer questions, both theoretical (e.g. epidemiology, pathophysiology) and with clinical application (e.g. case management, writing of prescriptions).

### Ethical considerations

All patients and health care providers gave their written informed consent to participate in the project, and also to appear on the Internet. All students gave their informed consent to participate in the evaluation. Ethical approval from the university or hospital was not required for this type of quality improvement project.

### Statistics

The standard deviations from the observed data of the NUDOV group and the non-NUDOV group were used as input in the power calculation. A difference of 5% in performance between the groups was assumed to be of importance. The beta (type II error) and alpha (type I error) values were set to 90% and 5%, respectively. To evaluate the effect of NUDOV on the examination result, a robust linear regression analysis was performed. There was no suspicion of any high leverage points (x-space) and therefore the M-estimator with a bisquare weight function was used to fit the regression line to the data. All analyses were performed using the procedures Power and Robustreg in the SAS software version 9.1.3 (SAS Institute, Inc., N.C., USA).

## Results

### Student questionnaires

All 31 NUDOV students completed their questionnaires. Twenty-eight of 31 (90%) NUDOV students stated that the program facilitated their learning to a large/very large degree. In particular, 26/31 (84%) perceived that their knowledge was achieved more rapidly than from conventional teaching only. The majority (22/31; 71%) reported that extensive working with authentic computerised cases made it easier to understand and learn about diseases and their management. Other major gains reported by the NUDOV students included the ability to work at their own pace, making it possible to repeat and reflect (20/31; 65%), which was not always possible when working with patients in a clinical setting. Further, NUDOV enabled several students (18/31; 58%) to encounter cases with important diagnoses, which not all had the possibility to meet elsewhere during the course. The majority were very pleased with the NUDOV layout (27/31; 87%), and its user friendliness and clarity (30/31; 97%). All reported the feedback concept, i.e. the comparison with what a specialist would do, to be good/very good. The perceived negative aspect of NUDOV was the requirement to write detailed patient records (8/31; 26%), and some students (5/31; 16%) found each case too time-consuming. Log files revealed that the students, in general, worked with each case for 60–90 (range 16 to 138) minutes.

### Learning outcome

**Figure 3:**
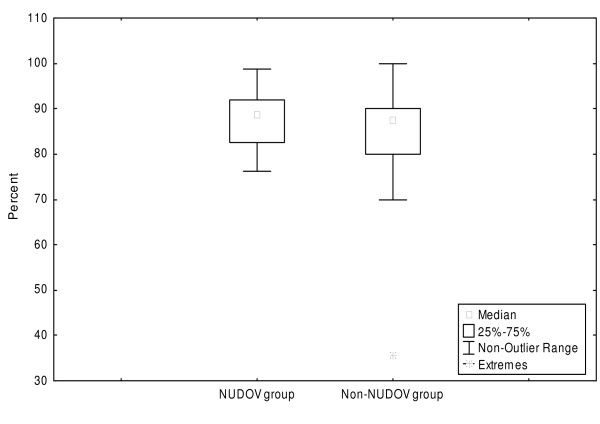
Percent correct answers of the NUDOV (n = 28) and the non-NUDOV (n = 81) groups in the final written examination for medical undergraduate students in dermatology and venereology.

Eighty-one of 85 (95%) non-NUDOV students and 28 of 31 (90%) NUDOV students participated in the written examination terminating each course. Seven students were ill. The NUDOV group scored better with higher percent correct answers than the non-NUDOV group did (median 88.8 percent units, interquartile range 9.38, versus median 87.5 percent units, interquartile range 10.0) (Fig. 3), but this difference was not statistically significant (mean difference 2.36 percent units [95% confidence interval -0.52; 5.26], p = 0.11).

## Discussion

The aim of the project was to develop and evaluate a national, interactive and computerised educational program in dermatology and venereology for undergraduate students in Swedish medical programmes. A short clinical course, such as the dermatology and venereology course, cannot guarantee that all students meet even the most common and important diagnoses. Therefore, we think that the learning system should be based on frequent and important authentic cases, so that every student will be able to obtain experience in both diagnosing and managing them.

The study questionnaire showed that the great majority of the NUDOV students appreciated the layout, concept and user friendliness of the program and that they considered that NUDOV facilitated their learning. Especially the feedback model where the student compares his or her suggestions with those of a specialist was highly appreciated, as reflected by the 100% positive answers. Yet the NUDOV students did not perform significantly better than the non-NUDOV students in the written final examination. This is in accordance with other studies, which also have indicated problems with detecting significant learning outcome improvements after e-learning interventions [13].

However, many factors may influence the evaluation of the effectiveness of educational interventions, such as the complex nature of education itself, sampling and outcome measures [14]. Choosing an adequate study methodology in educational research is not different from other type of research. In particular, two problems have to be addressed for our study: its power, and the traditional written examination as an objective outcome measure.

Given the sample sizes and variances of the present study, the power was 95% to detect a difference of 5 percent units between the NUDOV group and the non-NUDOV group. Such a difference was judged to be of interest.

The percent of correct answers in the examination may be a less ideal outcome measure. The written examination comprises questions where the students should give very short answers, e.g. stating of the most probable diagnosis for a clinical picture, mentioning of some theoretical facts (e.g. pathophysiology, epidemiology, clinical presentation etc.) or summarising the management of a disease or symptom. The examination does not test the ability to take a patient history or to examine a patient in such a way that a record and a management plan should be written. These are issues that NUDOV addresses. Nevertheless, a change in the examination design for the evaluation of NUDOV was considered too expensive and elaborate, involving also another teaching hospital. Today we believe that a different format of the assessment would have been appropriate and advisable. The present examination does not evaluate optimally the items that are in focus in NUDOV or in a real patient situation, and which we considered to be very important when we made the program. NUDOV aims at supporting students' problem-solving abilities and their clinical skills, and to provide a knowledge base of the most important disorders in the field in a way that supports a long-term approach of meaningful learning. The assessment procedure on the other hand follows the traditional way of measuring factual recall. Research on learning in higher medical education asserts that such an approach is problematic when assessing problem-solving and knowledge [15]. When evaluating whether a CAL tool will improve learning, it is important to use a valid assessment tool that measures the learning outcome [16]. In addition, it is well established that the examination has strong influence on what students learn [17-19]. An investigation of students' ability to investigate real patients, to suggest and discuss working diagnoses, differential diagnoses and management, and to write records/prescriptions, would have been a more appropriate test for the evaluation of the program.

Furthermore, it should be considered that with the high scores on this examination, it is possible that there was literally no room for detecting a significant improvement in the scores of the intervention group.

Research on how to evaluate computer innovations in learning highlights the problem of studies across different modes of instruction, such as the present one [20,21]. The question is perhaps not whether CAL per se is better than learning from traditional teaching, but rather how the design of software and its integration into the course interacts with the learning process.

Much time and resources were spent on development of the software itself. To fully benefit from the innovation, the implementation of the program into the curriculum deserves just as much attention and resources as the technical development. In addition to a suitable examination matching the outcome goals of the course, adjustment of the students' time schedule during the course and explaining to staff and students how to approach the tool are important. To avoid imposing an extra workload on the students, time to work with NUDOV has to be introduced into the students' schedule. The software is installed in our department's learning lab and CD-ROMs are also available for installing on the students' own computers should they wish to work with the program elsewhere.

## Conclusion

Although we have been unable to show that NUDOV significantly improves student performance in a traditional written examination, we consider the program to be a major achievement because of its: (1) reusability where the same authentic patients can be used over and over again, (2) structured and user-friendly software allowing an interactive non-linear use, (3) concept of learning about diseases and their management in a patient-oriented context, (4) feedback through comparison with a specialist, and (5) opportunity to analyse all student transactions through a log file. The flexible technical solution will enable us to gradually add modules with new cases and possibly also to move the system to the Web.

## Abbreviations

CAL Computer-assisted learning

NUDOV Nationellt Undervisningsprogram i Dermatologi Och Venereologi

## Competing interests

The author(s) declare that they have no competing interest.

## Authors' contributions

CFW, SE, UF and MS were all involved in creating the NUDOV program concept. CFW recruited the patients within it, took photos and wrote clinical texts. SE made the program under supervision of UF. HH and CFW designed and supervised the evaluation, which was carried out by CFW and SE. CFW and SE also drafted the manuscript. All authors were involved in revising it critically and approved the final version. MS and CFW were responsible for funding of the project.

## Pre-publication history

The pre-publication history for this paper can be accessed here:
